# Inappropriate prescribing of drugs for peptic ulcer and gastro-esophageal reflux disease remains a matter of concern: Results from the LAPTOP-PPI cluster randomized trial

**DOI:** 10.3389/fphar.2024.1430879

**Published:** 2025-01-03

**Authors:** Manuela Casula, Ilaria Ardoino, Laura Pierini, Lara Perrella, Stefano Scotti, Sara Mucherino, Valentina Orlando, Enrica Menditto, Carlotta Franchi

**Affiliations:** ^1^ Epidemiology and Preventive Pharmacology Service (SEFAP), Department of Pharmacological and Biomolecular Sciences, University of Milan, Milan, Italy; ^2^ IRCCS MultiMedica, Sesto San Giovanni, Milan, Italy; ^3^ Laboratory of Pharmacoepidemiology and Human Nutrition, Department of Health Policy, Istituto di Ricerche Farmacologiche Mario Negri IRCCS, Milan, Italy; ^4^ Department of Pharmacy, Center of Pharmacoeconomics and Drug Utilization Research (CIRFF), University of Naples Federico II, Naples, Italy

**Keywords:** proton pump inhibitors, inappropriate prescription, algorithm, pragmatic trial, informative intervention

## Abstract

**Background:**

Proton pump inhibitors (PPIs) are among the most commonly and inappropriately prescribed drugs by general practitioners (GPs), resulting in increased risk of adverse outcomes for patients and in avoidable costs for Italy’s National Health Service (NHS). This study aims to assess the effectiveness of a low-cost and easily implementable informative intervention directed at GPs to enhance the appropriate prescription of PPIs.

**Methods:**

The LAPTOP-PPI study is a pragmatic, cluster-randomized controlled trial designed to improve the appropriateness of PPI prescriptions among community-dwelling individuals aged ≥65 years. In June 2021, GPs in the Local Health Units (LHUs) of Bergamo (Northern Italy) and Caserta (Southern Italy) were randomly allocated to either an intervention group (summary reports on prescribing habits, scientific documents on the Italian Medicine Agency’s therapeutic indications, strategies for PPI de-prescribing, along with educational materials for patients), and a control group (standard practice). PPI appropriateness was assessed through an algorithm specifically designed and based on NHS prescription appropriateness and reimbursement criteria. Intervention efficacy was evaluated by comparing data from the baseline period (July 1 to 31 December 2019) with those from the follow-up period (July 1 to 31 December 2021), 6 months after randomization. The analysis was performed on the intention-to-treat principle and according to GP level. To estimate the effectiveness of the intervention, we used a difference-in-differences (DID) approach.

**Results:**

Overall, 942 GPs (540 from Bergamo and 402 from Caserta LHUs) were included in the analysis. At baseline, 171,978 patients aged ≥65 received drug prescriptions for acid-related diseases and were assessable for evaluation of appropriateness. At follow-up, this number was 137,699. The overall inappropriateness rate at baseline among GPs included in the analysis was 57.4% (std.dev. 8.4%) in the intervention arm and 57.6% (std.dev. 8.8%) in the control arm; 6 months after the intervention delivery, they were 59.2% (std.dev. 8.0%) and 58.5% (std.dev. 7.3%), respectively.

**Conclusion:**

Given their widespread use, improving the prescription quality of PPIs is a major concern. Educational interventions for GPs and patients are routine strategies to address inappropriateness, but they appear to be insufficient for independently improving prescribing practice, especially in a critical situation such as the post-pandemic period.

## 1 Introduction

Gastro-esophageal reflux disease (GERD) is prevalent on a global scale, affecting approximately 14% of the population—an estimated 1.03 billion individuals worldwide (Nirwan et al., 2020). Italy stands out among the countries with the highest GERD prevalence rates and significant utilization of related pharmacological treatments (Zhang et al., 2022). Management of GERD typically involves anti-acids, H2 receptor antagonists (anti-H2), proton pump inhibitors (PPIs), and prostaglandins (Gyawali and Fass, 2018). Currently, PPIs represent the mainstay of medical treatment for GERD; they are also employed for preventing drug-induced ulcers from antiplatelet or nonsteroidal anti-inflammatory drugs (NSAIDs), eradicating *Helicobacter pylori* (HP), and treating Zollinger–Ellison syndrome or Barrett’s esophagus (Scarpignato et al., 2016; Savarino et al., 2018). According to recent Italian data, the utilization of GERD medications in 2022 was 86.2 defined daily doses (DDDs)/1,000 inhabitants/day, with PPIs accounting for 90% of consumption, which represents a 1.9% increase from 2021 (AIFA, 2023).

Despite the established efficacy and tolerability of PPIs, evidence suggests that they are often inappropriately prescribed (Wallerstedt et al., 2017; Jaynes and Kumar, 2019; Franchi et al., 2020; Nguyen and Tamaz, 2018; Voukelatou et al., 2019; Lenoir et al., 2019), exposing patients to an increased risk of adverse drug reactions (ADRs) such as hypomagnesaemia, *Clostridium difficile* infection, pneumonia, chronic kidney disease, and fractures (Jaynes and Kumar, 2019). Inappropriate prescribing is of particular concern in older patients, who are often affected by multiple chronic diseases, exposed to polypharmacy, and are thus at further increased risk of potential drug–drug interactions (DDIs) and ADRs (Franchi et al., 2016; Franchi et al., 2019). Despite these risks, the use of GERD medications increases with age, peaking at 50% prevalence among patients aged 75 years or older (AIFA, 2023).

Over recent decades, both clinical trials and observational studies have prioritized the promotion of appropriate GERD drug prescriptions in different settings (Savarino et al., 2018; Franchi et al., 2020; Fan et al., 2023; Yailian et al., 2022; Veremme et al., 2021). In this context, we designed the pragmatic, cluster-randomized controlled trial “Evaluation of the effectiveness of a Low-cost informative intervention to improve the Appropriate PrescripTiOn of Proton PumP Inhibitors in older people in primary care: a cluster-randomized controlled study (LAPTOP-PPI).” We developed an easily implementable informative intervention targeted at general practitioners (GPs) operating in the Local Health Units (LHUs), with the aim of reducing the inappropriate prescription of GERD medications among community-dwelling, ≥65 individuals (Ardoino et al., 2022). Here, we present the main results of the pre-post analysis, assessing the effectiveness of our intervention on the rate of inappropriate prescription of drugs for GERD.

## 2 Materials and methods

### 2.1 Study design

LAPTOP-PPI was a pragmatic, cluster-randomized controlled trial (Clinicaltrial.gov: NCT04637750) addressed at GPs (clusters) of the Bergamo (northern Italy) and Caserta (southern Italy) LHUs between July and December 2019. The main aim was to reduce the rate of inappropriate drug prescription for peptic ulcer and GERD for older patients.

At baseline, all patients aged 65 years or over living in Bergamo and Caserta between July and December 2019 and receiving at least one drug prescription were scrutinized to assess the appropriateness of drug prescription for peptic ulcer and GERD, according to an algorithm based on administrative data and AIFA (Italian Medicine Agency) reimbursement criteria (Supplementary Figures S1, S2) (Ardoino et al., 2022).

At the end of the baseline period, GPs with at least one patient prescribed drugs for GERD were randomly assigned to the intervention or control arm. The randomization was performed centrally at the Istituto di Ricerche Farmacologiche Mario Negri IRCCS and was stratified according to the LHU. A complete randomized design was used. Two randomization lists were generated separately for the two LHUs using the Proc Plan procedure of SAS 9.4. GPs working within a group practice were randomized as a single unit.

The primary outcome of the trial was the change in appropriateness of the prescription of drugs for peptic ulcer and GERD assessed 6 months after intervention delivery, among patients receiving at least one prescription between July and December 2021.

### 2.2 Study intervention

In June 2021, all GPs received a letter illustrating the study purpose by their own local authority.

In addition, the GPs randomized to the intervention arm received a comprehensive package of resources and materials aimed at improving their prescribing practices, particularly with regard to the management of peptic ulcer disease and GERD. They were specifically provided with the following.

- A concise but detailed document designed to remind them of the AIFA reimbursement criteria for the appropriate prescription of drugs used to treat peptic ulcers and GERD. This document also included important information regarding the potential adverse effects associated with the long-term use of these medications in older patients. Furthermore, it offered practical suggestions and strategies for de-prescribing these drugs when appropriate, thus ensuring safer use among the elderly population.
- A personalized feedback report that provided insights into their prescribing patterns. This report contained the absolute number of their patients who had received at least one prescription for GERD medications, as well as the proportion of patients whose prescriptions were deemed inappropriate. This data was presented in comparison to the average prescribing practices observed within their local area, enabling the GPs to evaluate their performance relative to their peers.
- Information posters and leaflets intended for patient education. These materials served to raise awareness among patients about PPIs and the potential side effects associated with their prolonged use. In addition to risk information, these resources also offered practical nutritional and behavioral lifestyle advice for preventing gastric and reflux-related problems. Patients were also provided with guidance on how to safely reduce or discontinue the use of PPIs, facilitating a step-down approach to treatment when clinically appropriate.

This multifaceted intervention aimed to enhance the GPs’ knowledge, improve patient safety, and promote more appropriate prescribing practices through education, feedback, and patient engagement.

### 2.3 Data collection

Data for the analysis were obtained from the administrative databases of Bergamo and Caserta LHUs. All community-dwelling subjects aged 65 or over who received at least one prescription for a drug for peptic ulcer and GERD (ATC code: A02BA*-A02BB*-A02BC*) between July and December 2019 (baseline) or between July and December 2021 (6-month follow-up) were scrutinized and assessed for the study outcome.

The administrative databases gathered information for every patient assisted by the Italian National Health Service (NHS), collecting data on 1) sociodemographic characteristics; 2) drugs prescribed by the GPs and partially or entirely reimbursed by the NHS and dispensed through community pharmacies, 3) hospital discharge records reporting the main diagnosis, and up to five comorbidities, other than diagnostic and therapeutic procedures implemented during hospitalization; 4) outpatient services such as ambulatory specialist visits, diagnostic procedures, and laboratory tests that were provided by health providers accredited with the NHS; 5) national exemption codes that ascertain acute and chronic conditions for which patients were assisted free of charge [https://www.salute.gov.it/portale/esenzioni/dettaglioContenutiEsenzioni.jsp?lingua=italiano&id=1017&area=esenzioni&menu=vuoto] (Franchi et al., 2022). Trifiro et al. (2019) describe the structure of these databases in more detail. Drug prescriptions were codified according to the Anatomical Therapeutic Chemical (ATC) classification system. Comorbidities were identified by International Classification of Diseases, Ninth Revision, Clinical Modification (ICD-9- CM) classification. Record linkage between different sources was facilitated by a national unique identification code assigned to each patient. Identification codes were automatically converted into anonymized codes, and the conversion table was stored by the regional authorities which were overseeing data.

### 2.4 Algorithm for prescription inappropriateness

Prescription inappropriateness of GERD medications was established according to AIFA reimbursement rules, NOTAs 1 and 48 (Ardoino et al., 2022) (Table 1). NOTA 1 allows prescription for the prevention of serious complications of the gastrointestinal tract in patients at high risk who are chronically prescribed with low dose acetylsalicylic acid (ASA) or NSAIDs with specific requirements. NOTA 48 allows short term prescription (up to max. 6 weeks) for the treatment of first episode or recurrent duodenal or gastric ulcer (positive or negative to *H. pylori*).

**TABLE 1:** Rules of the Italian Medicines Agency (AIFA) for the reimbursement of PPI and other drugs for GERD.

NOTA 1
Reimbursement from the National Health Service of prescriptions of PPIs and misoprostol is limited to the prevention of serious complications of the upper gastrointestinal tract in patients on chronic treatment with NSAIDS or on antiplatelet therapy with low doses of ASA for cerebro- or cardio-vascular prevention, provided there is one of the following condition risks: • History of past digestive hemorrhages or peptic ulcer • Concomitant therapy with anticoagulants or corticosteroids • Advanced age

NOTA 48
Reimbursement of PPIs and H2-receptor antagonist prescription by the National Health Service is limited to the following periods and conditions **-** Duration of treatment of 4 weeks (occasionally 6 weeks) - Duodenal or gastric ulcer, in association with drugs that eradicate the infection - GERD with or without esophagitis (first episode) - Extended duration of treatment, to re-evaluate after 1 year - Zollinger–Ellison syndrome - Relapsing duodenal or gastric ulcer - GERD with or without esophagitis (relapsing)

PPIs, proton pump inhibitors; GERD, gastro-esophageal reflux disease; ASA, acetylsalicylic acid; NSAIDS, non-steroidal anti-inflammatory drugs.

Patients were classified as occasional, short-term, or chronic users. Occasional users had one prescription for GERD treatment lasting no more than 28 days during the baseline period. Short-term users received treatment for 29–60 days, while chronic users had more than 60 days of treatment. Occasional users were considered appropriately prescribed, regardless of compliance with AIFA NOTAs, as they may have been prescribed on-demand for symptom relief. Prescription appropriateness was assessed for the remaining patients according to AIFA NOTA 1 and then NOTA 48 criteria (Supplementary Figures S1, S2). Using the same algorithm, we also evaluated patients who would have had an indication of treatment but were not prescribed with drugs for GERD.

### 2.5 Statistical methods

#### 2.5.1 Sample size

We expected to include nearly 800 GPs accounting on average for 200 older patients prescribed with drugs for GERD. This should have allowed detection of an expected reduction of inappropriate prescription rates ranging from 10% to 20% in the intervention group, and one of 2% in the control arm for the effect of participation in the study with a power of 98%.

#### 2.5.2 Statistical analysis

The analysis was performed on the intention-to-treat principle and according to GP level. To estimate the effectiveness of the intervention, we used a difference-in-differences (DID) approach. This allowed for the comparison of the changes in an outcome between the pre (baseline) and post periods (6 months after intervention delivery) in the intervention and control arms, and then subtracted one from the other to identify the “difference in the differences” between the groups (Warton et al., 2016). According to the aim of this study, we used a repeated measure logistic regression model on the proportion of subjects appropriately prescribed for each GP with an identity link function, accounting for the correlation between measures within the GP, adjusting for time (pre-post period), exposure (intervention-control arm), and the interaction between time and exposure. Regression analysis was further adjusted for GPs’ main characteristics (sex, age, and number of patients or of older patients in charge).

## 3 Results

At baseline (from July to December 2019), Bergamo and Caserta LHUs accounted for 628 and 583 GPs, respectively. Among 628 GPs in Bergamo LHU, 180 (28.7%) worked alone and 448 (71.3%) worked within a group practice, making a total of 295 groups. In Bergamo LHU, 314 GPs were randomized to the intervention arm and 314 to the control arm, and in Caserta LHU, 294 GPs were randomized to intervention and 289 to the control arm.

The main characteristics of the randomized GPs, according to intervention arm and geographical area, are reported in Table 2. Among GPs from Bergamo LHU, 240 were female (38.2%) and the mean age was 58.0 (std.dev. 8.5) years. In Caserta LHU, proportion of female GPs was lower (N = 144, 24.7%) and the mean age was slightly older (63.8, std. dev. 4.5 years).

**TABLE 2:** Main characteristics of 628 GPs in Bergamo and 583 GPs in Caserta randomized at the end of the baseline period (July–December 2019).

	Intervention		Control	
	Bergamo	Caserta	Bergamo	Caserta
Number of GPs	314 (50%)	294 (50.4%)	314 (50%)	289 (49.6%)
Age—mean (s.d.)	57.5 (8.7)	62.7 (4.7)	58.4 (8.3)	62.9 (4.4)
Male—number (%)	192 (61.2%)	221 (75.2%)	196 (62.4%)	218 (75.4%)
Patients in charge—median [IQR]	1,531.5 [1,426; 1,570]	1,478.5 [1,196; 1,560]	1,527 [1,340; 1,557]	1,505 [1,154; 1,564]
Older patients in charge—median [IQR]	360.5 [298; 417]	290.5 [218; 343]	362 [298; 405]	280 [204; 341]
Patients prescribed with drugs for peptic ulcer or GERD—median [IQR]	127 [100; 154]	172 [123; 207]	133 [102; 158]	165 [117; 200]
Patients with indication but not prescribed with drugs for peptic ulcer or GERD—median [IQR]	227 [187; 257]	113 [82; 148]	220 [181; 266]	111 [71; 144]

GPs, general practitioners; IQR, interquartile range; s.d., standard deviation.

**FIGURE 1:**
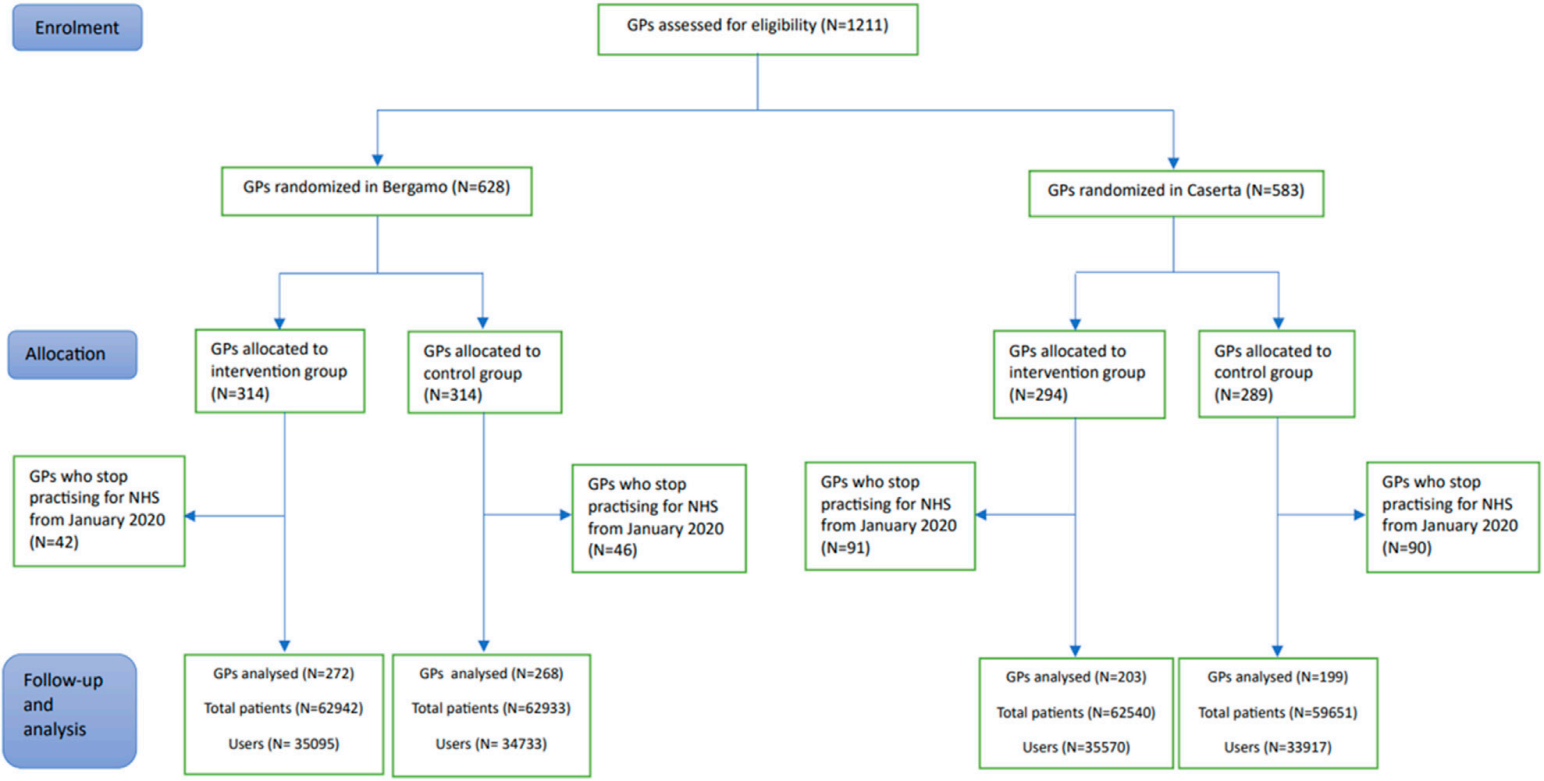
Flow-chart describing the number of General Practitioners (GPs) included in the enrolment, allocation, follow-up, and analysis phases of the LAPTOP-PPI trial.

From 2020 onward, 88 GPs (14.0%) of Bergamo and 181 GPs (31.0%) of Caserta LHUs had stopped working for the NHS, so that 942 GPs (540 from Bergamo and 402 from Caserta LHUs) were included in the analysis. In Bergamo LHU, 272 GPs (50.4%) were randomized to the intervention arm and 268 GPs (49.6%) to control. Among them, 21 GPs (11 in the intervention arm and 10 in the control arm) did not prescribe drugs for GERD to their patients at 6 months after intervention delivery and thus were not assessable at follow-up. In Caserta LHU, 203 (50.5%) were in the intervention arm while 199 (49.5%) were in control (Figure 1).

The 942 GPs included in the analysis had overall charge of 194,173 subjects in Bergamo LHU and 129,479 in Caserta LHU aged 65 years or over and receiving at least one drug prescription. Among these, 137,699 patients were prescribed at least once with drugs for peptic ulcer or GERD, so that 69,266 (35.7%) in Bergamo LHU and 67,871 (52.4%) in Caserta LHU and were eligible for the present analysis.

Results of the main outcome for the two LHUs are reported in Table 3. The overall inappropriateness rate at the baseline among GPs included in the analysis was 57.4% (std.dev. 8.4%) in the intervention arm and 57.6% (std.dev. 8.8%) in the control arm; 6 months after the intervention delivery, they were 59.2% (std.dev. 8.0%) and 58.5% (std.dev. 7.3%), respectively. In Bergamo LHU, at baseline, the average inappropriateness rate among GPs was 53.5% (std.dev. 7.1%). After the intervention period, it became 58.3% (std.dev. 8.6%)—59.0% (std.dev. 9.1%) in the intervention arm and 57.6% (std.dev. 8.0%) in the control arm. In Caserta LHU, at baseline the average inappropriateness was 62.8% (std.dev. 7.5%), becoming 59.5% (std.dev. 6.2%) after 6 months—59.3% (std.dev. 6.3%) in the intervention arm and 59.7% (std.dev. 6.1%) in the control arm. Significant differences among baseline and follow-up were found, with a slight increase in overall inappropriateness in Bergamo (+4.3%) and a slight decrease in Caserta (−3.4%), while no difference was found between the intervention and control arms. Supplementary Tables S1 and S2 show detailed results on the appropriateness criteria of drugs for GERD prescription for the two LHUs according to intervention arm. When a logistic regression adjusted model was fitted, no GP-related characteristics were found to be associated with the outcome (data not shown).

**TABLE 3:** Primary outcome for GPs randomized and present at 6 months follow-up (included in the analyses).

	Intervention		Control	
	Number of GPs	Inappropriateness rate – mean (s.d.)	Number of GPs	Inappropriateness rate – mean (s.d.)
Bergamo				
Baseline	272	53.6 (7.1)	268	53.5 (7.1)
After 6 months FU	261	59.0 (9.1)	258	57.6 (8.0)
Caserta				
Baseline	203	62.4 (7.3)	199	63.3 (7.7)
After 6 months FU	203	59.3 (6.3)	199	59.7 (6.1)
Total				
Baseline	475	57.4 (8.4)	467	57.6 (8.8)
After 6 months FU	464	59.2 (8.0)	457	58.5 (7.3)

GPs, general practitioners; IQR, interquartile range; s.d., standard deviation.

## 4 Discussion

### 4.1 Discussion of trial results

Drugs for GERD, particularly PPIs, are among the most consumed drug classes, and are also frequently associated with high inappropriate prescription rates. Despite their efficacy and generally favorable safety profile demonstrated in numerous clinical trials (Malhotra et al., 2018), medium- to long-term PPI use has been linked to various adverse effects. This concern is exacerbated by the fact that this widespread use is often practiced in the absence of a real indication for treatment or is prolonged for longer than necessary. Our study found that, in two Italian LHUs, more than one in two prescriptions for GERD drugs to patients over 65 did not meet the appropriateness criteria set by the national drug authority. This discouraging picture did not seem to be changed by the information intervention implemented in our trial, with no significant differences in the pre–post evaluation between intervention and control arms.

To comprehend what may have led to this result, two components should be distinguished. On the one hand, we should try to understand why the implemented intervention did not yield the desired result. On the other hand, it is crucial to analyze the context, which suggests a secular trend toward a worsening of the inappropriateness rate, which may have minimized the impact of the intervention.

In some cases, educational interventions for GPs have proven effective in changing PPIs prescribing practice (Del Giorno et al., 2018; Walker et al., 2019). A very similar study (Casula et al., 2016), designed to improve treatment adherence in patients starting statin treatment, showed that an informative intervention on Italian GPs (specifically, a personalized document reporting aggregated data on adherence in 2006 for each GP’s patients compared to the means in the LHU) was able to significantly decrease the proportion of patients dropping out of treatment after only one prescription, and to increase the mean adherence and duration of continued therapy. Hallsworth et al. (2016) implemented a pragmatic randomized controlled trial to reduce unnecessary prescriptions of antibiotics by GPs in England. GPs in the intervention arm received a letter reporting the rate of antibiotic prescribing compared to the local area performance, and this led to a substantial reduction in antibiotic prescribing.

However, in other cases, this approach has failed to change the prescribing habits of doctors, and this seems particularly true for studies that specifically target PPI prescription. For example, van Vliet et al. (2009) designed a study to assess whether implementing a guideline for PPI prescription in pulmonary medicine wards could lead to a decrease in use and improved appropriateness of prescription. After the intervention, fewer patients were started on PPIs and more users discontinued their use; however, the appropriateness of prescribing PPIs was not affected. Jain et al. (2013) found that physicians largely prescribed drugs for stress ulcer prophylaxis, despite demonstrating good knowledge of treatment guidelines. Moreover, following an intervention to decrease the inappropriate use of these treatments, prescription rates decreased significantly while the rate of inappropriate continuation of acid suppressive therapy did not change over the study period. In Lazaridis et al. (2021), an educational intervention in a Greek university hospital failed to reduce the inappropriate use of PPIs during hospitalization or at discharge in the internal medicine patients. Recently, in Muskens et al. (2024), an educational intervention using waiting room posters and flyers aimed at both patients and GPs regarding the appropriate indications for acid-reducing medications did not result in a change in their chronic prescription.

It has been consistently demonstrated that influencing the prescribing habits of doctors is an incredibly challenging task, and many interventions aimed at achieving this have met with limited success (Galimberti et al., 2022; Giguere et al., 2020). Research has highlighted that approaches that tend to be more effective typically involve reminder systems, academic detailing (where experts engage with physicians one-on-one to provide targeted education), and the implementation of multiple, combined interventions rather than single strategies (Davis and Taylor-Vaisey, 1997). In particular, multifaceted interventions appear to address the complexity of clinical decision-making better, as they provide ongoing support and reinforcement to doctors. However, a survey conducted at Vancouver General Hospital examined the self-reported usefulness of educational resources provided during a 2-month intervention designed to improve PPI prescribing practices (Wan et al., 2018). Despite the availability of these resources, only 52% of respondents felt that the materials had a meaningful impact on their clinical practice. This suggests that even when educational resources are readily accessible, they may not always translate into changes in prescribing behavior, likely due to a variety of barriers.

One of the key reasons for this resistance to change is the fact that many physicians prioritize the management of their patients’ immediate symptoms over concerns about the long-term risks associated with inappropriate prescriptions. Studies have shown that doctors often seem more concerned about failing to control patients’ symptoms, however mild, or about addressing their complaints than they are about the potential adverse effects or toxicities of prolonged therapy (Wermeling et al., 2014; Grime et al., 2001). This highlights a tendency to favor short-term symptom relief over guideline-concordant prescribing, even when evidence suggests that such an approach could lead to harmful outcomes in the long run.

Moreover, the phenomenon of clinical inertia has been widely reported in the literature, particularly in general practice. “Clinical inertia” refers to a failure to modify treatment plans in accordance with current guidelines, even when evidence suggests that a change is necessary (Phillips et al., 2001; Roumie et al., 2007). This inertia has been documented in the context of various therapeutic areas, including the overuse of antacid medications like PPIs (Roumie et al., 2007). Factors that contribute to clinical inertia include a reluctance to discontinue or adjust treatment regimens, the perception that a patient’s current treatment is working satisfactorily, or a lack of awareness or familiarity with updated guidelines.

Finally, it is worth noting that the educational tools and materials utilized in our intervention are commonly employed by LHUs for routine educational and training purposes. Given their widespread and repeated use, it is plausible that some physicians in our study may have perceived the resources as redundant or unremarkable, particularly if they were already accustomed to receiving similar inputs in their day-to-day professional environment (Galimberti et al., 2022). This familiarity may have contributed to the underutilization of the intervention materials, especially given the voluntary nature of our study, where engagement with the resources was not mandatory. Therefore, the effectiveness of our intervention may have been limited by these contextual factors, thus reflecting broader challenges faced by similar interventions aimed at modifying prescribing behaviors in real-world clinical practice.

The other aspect that must be considered in the critical interpretation of our results is the pandemic context. The use of PPIs and the rate of inappropriate prescriptions have been shown to be increasing over time (Muheim et al., 2021). This trend may have been further exacerbated by the COVID-19 pandemic (AIFA, 2023; Suzuki et al., 2023). Prescriptions of these drugs may have increased not only in response to the growing need of the population, which was faced with a particularly stressful event; the significant increase in hospitalizations for COVID-related causes (particularly in Bergamo LHU) may have led physicians to intensify at-discharge prescribing for precautionary purposes, paying less attention to the real need for treatment.

Regarding the two LHUs involved, the rate of inappropriateness increased by about five percentage points in Bergamo and decreased by approximately four percentage points in Caserta. Evaluating each reimbursability criterion according to AIFA notes, one possible reason for this different trend seems to be the chronic consumption of ASA/NSAIDs and other anticoagulants at follow-up compare to baseline (Ardoino et al., 2022). In fact, it was approximately stable for Bergamo but strongly increased for Caserta. This is confirmed by regional data on drug consumption (AIFA, 2023). In Lombardy (Bergamo), the consumption of anticoagulants is described as fairly stable in the last decade, with a change of −1.6% in consumption in DDD/1,000 inhabitants/day between 2019 and 2021. In Campania (Caserta), the consumption trend has grown steadily since 2014, with a change of +5% in DDD/1,000 inhabitants/day between 2019 and 2021.

### 4.2 Strengths and limitations

The use of health administrative databases is certainly a strength of the study, as they collect all the reimbursed drugs dispensed to all citizens covered by the NHS. Administrative data collection, managed at a regional level, is nationally standardized, extremely accurate, and commonly used for drug utilization research (Trifiro et al., 2019). However, they do not contain clinical information such as diagnosis, indication for treatment, and results of examinations and tests. This may certainly have had an impact on the point estimate of inappropriateness, as it did not allow for a comprehensive characterization of the patient and led to a potential overestimation of the proportion of subjects without an indication for treatment. However, it is unlikely to have had a differential impact on the analysis at baseline and at follow-up, so we can consider the results of the trial to be robust.

The challenge in demonstrating the effectiveness of interventions in pragmatic studies is partly inherent in their design. Unlike controlled explanatory studies, pragmatic trials do not allow for strict control over the real-world application of interventions. Active participation could not be enforced, and there was no mechanism to verify whether GPs had indeed engaged with or considered the intervention materials, making it difficult to ascertain behavioral changes. Furthermore, the way the material was distributed did not allow us to know whether the doctor opened or read the e-mail.

Another major, though not avoidable, limitation of our study is the concomitance with the pandemic period. In 2020–2021, health priorities in Italy were largely governed by pandemic management and prevention activities through vaccination campaigns. For this reason, it is difficult to assess the effectiveness of a training intervention aimed at physicians in this context, and the result obtained is probably not generalizable to normal healthcare situations in the non-emergency period.

### 4.3 Conclusion

Our study provides some issues to consider. We are faced with a situation—the high rate of inappropriate prescription of drugs for GERD—which is extremely difficult to change. On the other hand, change is necessary to protect the health of patients and to contain healthcare costs. Next to educational paths, which can be implemented for the young generation of health professionals and will have long-term results, it is now urgent to find alternative strategies that can effectively guide physicians in prescribing these drugs. Having ascertained the limited impact of the strategy proposed by this study, new efforts are needed to exploit recent technologies and support GPs with user-friendly information tools and possibly interfacing with software already in routine use.

## Data Availability

The raw data supporting the conclusions of this article will be made available by the authors without undue reservation.
