# Supplementary material for: Inappropriate prescribing of drugs for peptic ulcer and gastro-esophageal reflux disease remains a matter of concern: Results from the LAPTOP-PPI cluster randomized trial
Source: Front Pharmacol. 2025 Jan 3;15:1430879. doi: 10.3389/fphar.2024.1430879 (PMC11739113; doi:10.3389/fphar.2024.1430879)
Supplement: Supplementary file 1 [file DataSheet1.docx]

Supplementary Material

# Supplementary Data

# Appropriateness among patients of Bergamo and Caserta, separated for control and intervention group, in charge of analysed General Practitioners (GPs)

# Among 628 GPs in Bergamo who were randomized, at the end of baseline (from July to December 2019), 540 were present after 6-month follow-up. The 272 GPs randomized in the intervention arm had in charge 34768 patients, while 268 GPs randomized in the control one had in charge 34498 patients. Among the first, 14482 (41.7%) patients were appropriately prescribed while among the last those appropriately prescribed were 14490 (42.0%). The same prevalences at the baseline were about 46.0% in both groups.

# Among the 583 GPs in Caserta, who were randomized, 402 were analysed at the end of the 6-month follow-up. The 203 GPs randomized in the intervention arm had in charge 34774 patients, while 199 GPs randomized in the control one had in charge 33097 patients prescribed with drugs for GERD and then assessable for appropriateness. Appropriate prescriptions were found for 14058 patients (40.4%) among GPs randomized in the intervention arm and for 13329 patients (40.2%) among GPs in the control one. At the baseline the same proportion were 37.2% and 36.9% respectively.

# Supplementary Figures

**Figure S1: Algorithm to assess appropriateness of Proton Pump Inhibitor prescriptions according to the reimbursement criteria of AIFA NOTE 1**


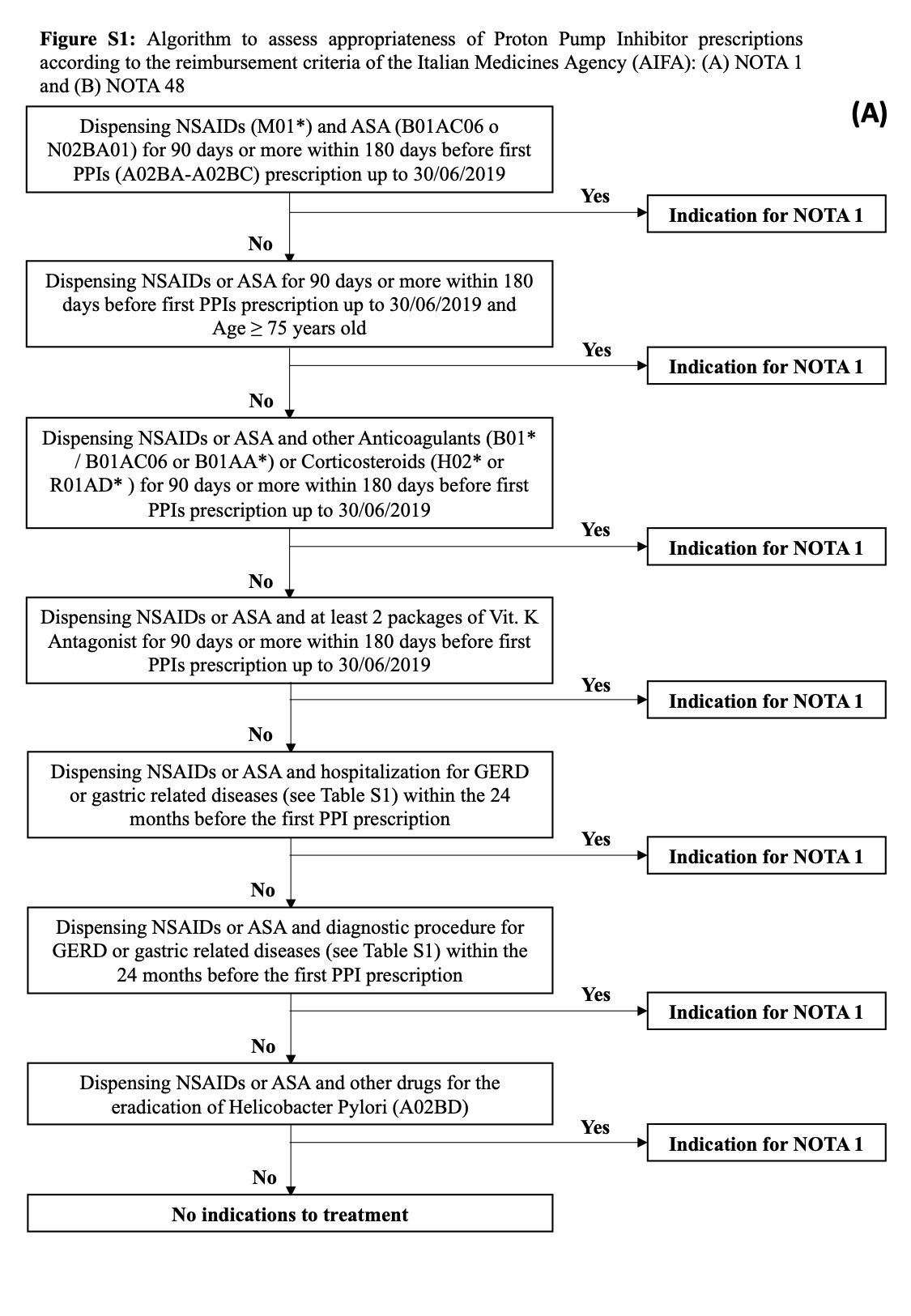


ASA: acetylsalicylic acid (aspirin); GERD: gastroesophageal reflux disease; NSAIDS: non-steroidal anti-inflammatory drugs

**Figure S2: Algorithm to assess appropriateness of Proton Pump Inhibitor prescriptions according to the reimbursement criteria of AIFA NOTE 48**


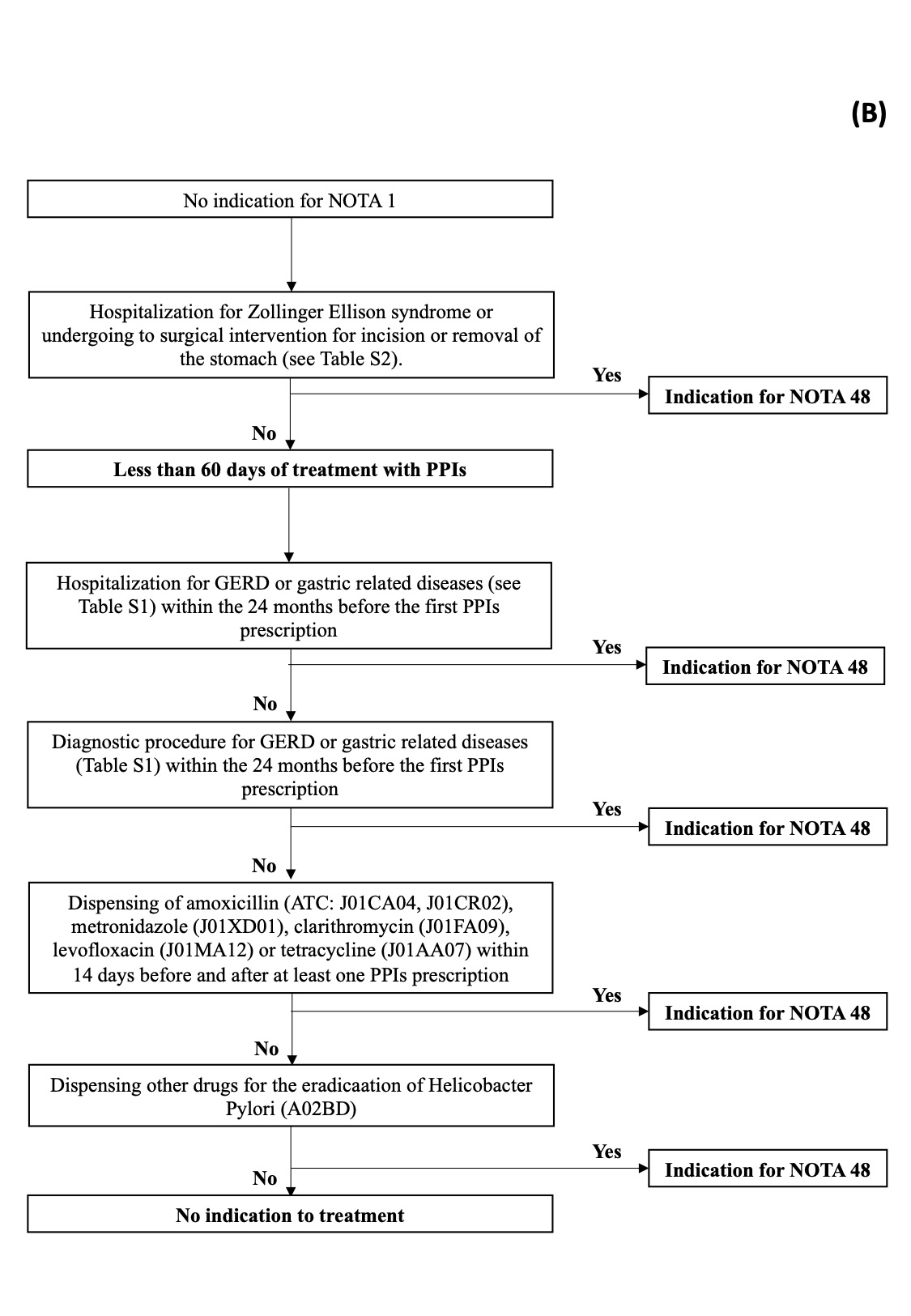


GERD: gastroesophageal reflux disease

# Supplementary Tables

**Table S1: Detailed appropriateness criteria at follow-up according to intervention arm - Bergamo**

|  | Intervention  (N=34768) | | Control  (N=34498) | |
| --- | --- | --- | --- | --- |
| Appropriate | 14482 (41.7%) |  | 14490 (42.0%) |  |
|  |  |  |  |  |
| Occasional |  | 3648 |  | 3882 |
|  |  |  |  |  |
| Appropriated for Nota1 |  | 9773 |  | 9624 |
| ASA & NSAIDs |  | 626 |  | 675 |
| Old (≥ 75 years) |  | 7467 |  | 7285 |
| Corticosteroids |  | 498 |  | 522 |
| Other Anticoagulants |  | 1952 |  | 1975 |
| Vit. K Antagonist |  | 321 |  | 361 |
| GERD |  | 3141 |  | 3090 |
| HP (co-prescription with combinations) |  | 2 |  | 5 |
|  |  |  |  |  |
| Appropriated for Nota48 |  | 2133 |  | 2171 |
| Zollingher-Ellison Syndrome |  | 260 |  | 258 |
| GERD |  | 1869 |  | 1925 |
| HP (co-prescription with combinations) |  | 8 |  | 8 |
| Suspected HP (co-prescription with ≥2 antibiotics) |  | 62 |  | 55 |
|  |  |  |  |  |
| Not Appropriate | 20286 (58.3%) |  | 20008 (58.0%) |  |

ASA: acetylsalicylic acid (aspirin); GERD: gastroesophageal reflux disease; GPs: general practitioners; HP: Helicobacter pylori; NSAIDS: non-steroidal anti-inflammatory drugs

**Table S2: Detailed appropriateness criteria at follow-up according to intervention arm – Caserta**

|  | Intervention  (N=34774) | | Control  (N=33097) | |
| --- | --- | --- | --- | --- |
| Appropriate | 14058 (40.4%) |  | 13329 (40.3%) |  |
|  |  |  |  |  |
| Occasional |  | 4416 |  | 4052 |
|  |  |  |  |  |
| Appropriated for Nota1 |  | 10012 |  | 9574 |
| ASA & NSAIDs |  | 2794 |  | 2710 |
| Old (≥ 75 years) |  | 7646 |  | 7274 |
| Corticosteroids |  | 420 |  | 430 |
| Other Anticoagulants |  | 3168 |  | 3072 |
| Vit. K Antagonist |  | 57 |  | 58 |
| GERD |  | 891 |  | 822 |
| HP (co-prescription with combinations) |  | 5 |  | 1 |
|  |  |  |  |  |
| Appropriated for Nota48 |  | 391 |  | 390 |
| Zollingher-Ellison Syndrome |  | 3 |  | 5 |
| GERD |  | 261 |  | 250 |
| HP (co-prescription with combinations) |  | 0 |  | 0 |
| Suspected HP (co-prescription with ≥2 antibiotics) |  | 137 |  | 144 |
|  |  |  |  |  |
| Not Appropriate | 20716 (59.6%) |  | 19768 (59.7%) |  |

ASA: acetylsalicylic acid (aspirin); GERD: gastroesophageal reflux disease; GPs: general practitioners; HP: Helicobacter pylori; NSAIDS: non-steroidal anti-inflammatory drugs
